# Combined *in Vitro* and *in Silico* Studies for the Anticholinesterase Activity and Pharmacokinetics of Coumarinyl Thiazoles and Oxadiazoles

**DOI:** 10.3389/fchem.2018.00061

**Published:** 2018-03-26

**Authors:** Aliya Ibrar, Ajmal Khan, Majid Ali, Rizwana Sarwar, Saifullah Mehsud, Umar Farooq, Syed M. A. Halimi, Imtiaz Khan, Ahmed Al-Harrasi

**Affiliations:** ^1^Department of Chemistry, Abbottabad University of Science & Technology, Havelian, Pakistan; ^2^Department of Chemistry, COMSATS Institute of Information Technology, Abbottabad, Pakistan; ^3^UoN Chair of Oman's Medicinal Plants and Marine Natural Products, University of Nizwa, Nizwa, Oman; ^4^Department of Pharmacy, University of Peshawar, Peshawar, Pakistan; ^5^Department of Chemistry, Quaid-i-Azam University, Islamabad, Pakistan; ^6^School of Chemistry, Cardiff University, Cardiff, United Kingdom

**Keywords:** coumarin thiazoles, coumarin oxadiazoles, cholinesterase inhibition, molecular docking, MOE score

## Abstract

In a continuation of our previous work for the exploration of novel enzyme inhibitors, two new coumarin-thiazole **6(a–o)** and coumarin-oxadiazole **11(a–h)** hybrids have been designed and synthesized. All the compounds were characterized by ^1^H- and ^13^C-NMR spectroscopy and elemental analysis. New hybrid analogs were evaluated against acetylcholinesterase (AChE) and butyrylcholinesterase (BuChE) in order to know their potential for the prevention of Alzheimer's disease (AD). In coumarinyl thiazole series, compound **6b** was found as the most active member against AChE having IC_50_ value of 0.87 ± 0.09 μM, while the compound **6j** revealed the same efficacy against BuChE with an IC_50_ value of 11.01 ± 3.37 μM. In case of coumarinyl oxadiazole series, **11a** was turned out to be the lead candidate against AChE with an IC_50_ value of 6.07 ± 0.23 μM, whereas compound **11e** was found significantly active against BuChE with an IC_50_ value of 0.15 ± 0.09 μM. To realize the binding interaction of these compounds with AChE and BuChE, the molecular docking studies were performed. Compounds from coumarinyl thiazole series with potent AChE activity (**6b**, **6h**, **6i**, and **6k**) were found to interact with AChE in the active site with MOE score of −10.19, −9.97, −9.68, and −11.03 Kcal.mol^−1^, respectively. The major interactions include hydrogen bonding, π-π stacking with aromatic residues, and interaction through water bridging. The docking studies of coumarinyl oxadiazole derivatives **11(a–h)** suggested that the compounds with high anti-butyrylcholinesterase activity (**11e**, **11a**, and **11b**) provided MOE score of −9.9, −7.4, and −8.2 Kcal.mol^−1^, respectively, with the active site of BuChE building π-π stacking with Trp82 and water bridged interaction.

## Introduction

**Figure 1:**
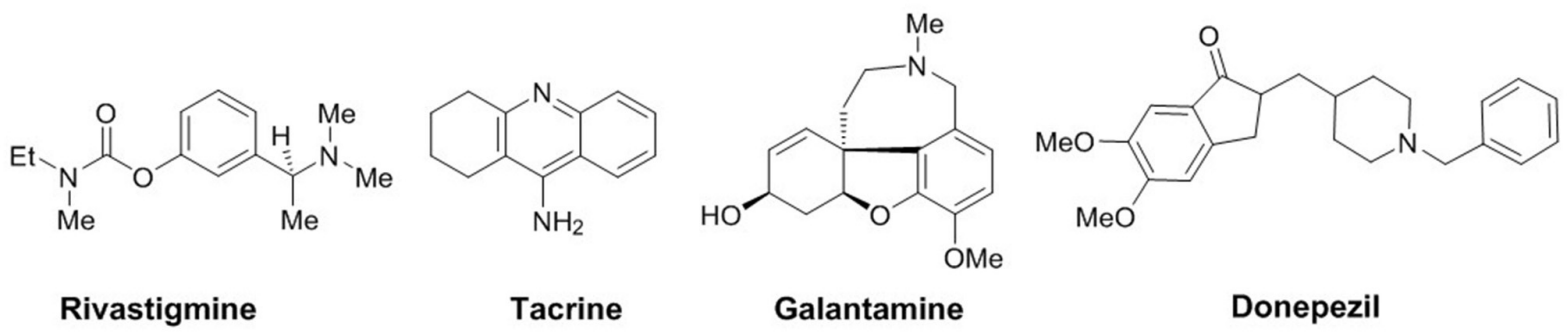
Cholinesterase inhibitors used in AD therapy.

Alzheimer's disease (AD), the most common cause of dementia, is a neurodegenerative disorder mainly characterized by progressive deterioration of memory and cognition (Terry and Buccafusco, 2003). One of the key therapeutic strategies adopted for primarily symptomatic AD is based on the cholinergic hypothesis targeting cholinesterase enzymes (acetylcholinesterase and butyrylcholinesterase; Cummings et al., 2007), two important enzymes from the group of serine hydrolases. Structurally, these serine hydrolases belong to the class of proteins known as the esterase/lipase family within the α/β-hydrolase fold superfamily (Cygler et al., 1993). The major role of AChE is the inhibition of the hydrolysis of acetylcholine in cholinergic synapses. Thus, blocking its metabolic activity and increasing the ACh concentration ultimately leading to a possible symptomatic treatment option for AD, whereas, the functional activity of butyrylcholinesterase (BChE) is less understood because it can hydrolyze ACh as well as other esters (Groner et al., 2007; Chiou et al., 2009). Butyrylcholinesterase has recently been considered as a potential target because it also plays an important role in regulating ACh level (Mesulam et al., 2002). AChE inhibitors currently approved as drugs for the treatment of Alzheimer's disease are donepezil, rivastigmine, galantamine, and tacrine (Figure 1). Although, donepezil is most commonly used AChE inhibitor, its Aβ formation inhibition activity is weak (Bartolini et al., 2003). In view of the limited number of cholinesterase inhibitors currently available for the treatment of AD, the search for new and potent inhibitors is of significant interest and a progressive area of current research.

Among oxygenated heterocycles, coumarin compounds have sustained efficacy as they inhibit both acetyl- and butyrylcholinesterase enzymes and help to slow down the formation of amyloid compounds (de Souza et al., 2016). Coumarins, both natural and synthetic demonstrate a wide spectrum of biological functions as they offer a wide range of structural changes on benzopyran ring. Activities like anti-tubercular (Manvar et al., 2011), anti-tumor (Maddi et al., 2007), anti-HIV (Kashman et al., 1992), anti-inflammatory (Ronad et al., 2010), anti-cancer (Olmedo et al., 2012), and anticoagulant (Martin-Aragón et al., 2001) have been reported. In addition, thiazole and oxadiazole skeletons are fundamentally important and versatile structural analogs of five-membered heterocyclic compounds. They show a vast majority of biological activities (Klimesová et al., 2004; Hang and Honek, 2005; Campiglia et al., 2009; Siddiqui et al., 2009; Jaishree et al., 2012; Romagnoli et al., 2012; Helal et al., 2013; Naveena et al., 2013; Venugopala et al., 2013; Yavari et al., 2014) in addition to be a part of numerous complex natural products like vitamin B1, penicillin (Shaker, 2006), and thiamine pyrophosphate, an important co-enzyme.

In the present study, two new coumarin-thiazole **6(a–o)** and coumarin-oxadiazole **11(a–h)** hybrids were synthesized and evaluated for their acetylcholinesterase (AChE) and butyrylcholinesterase (BuChE) inhibitory activity. Furthermore, the molecular docking studies on both series were also performed to explore their binding interactions.

## Results and discussion

### Chemistry

**Scheme 1:**
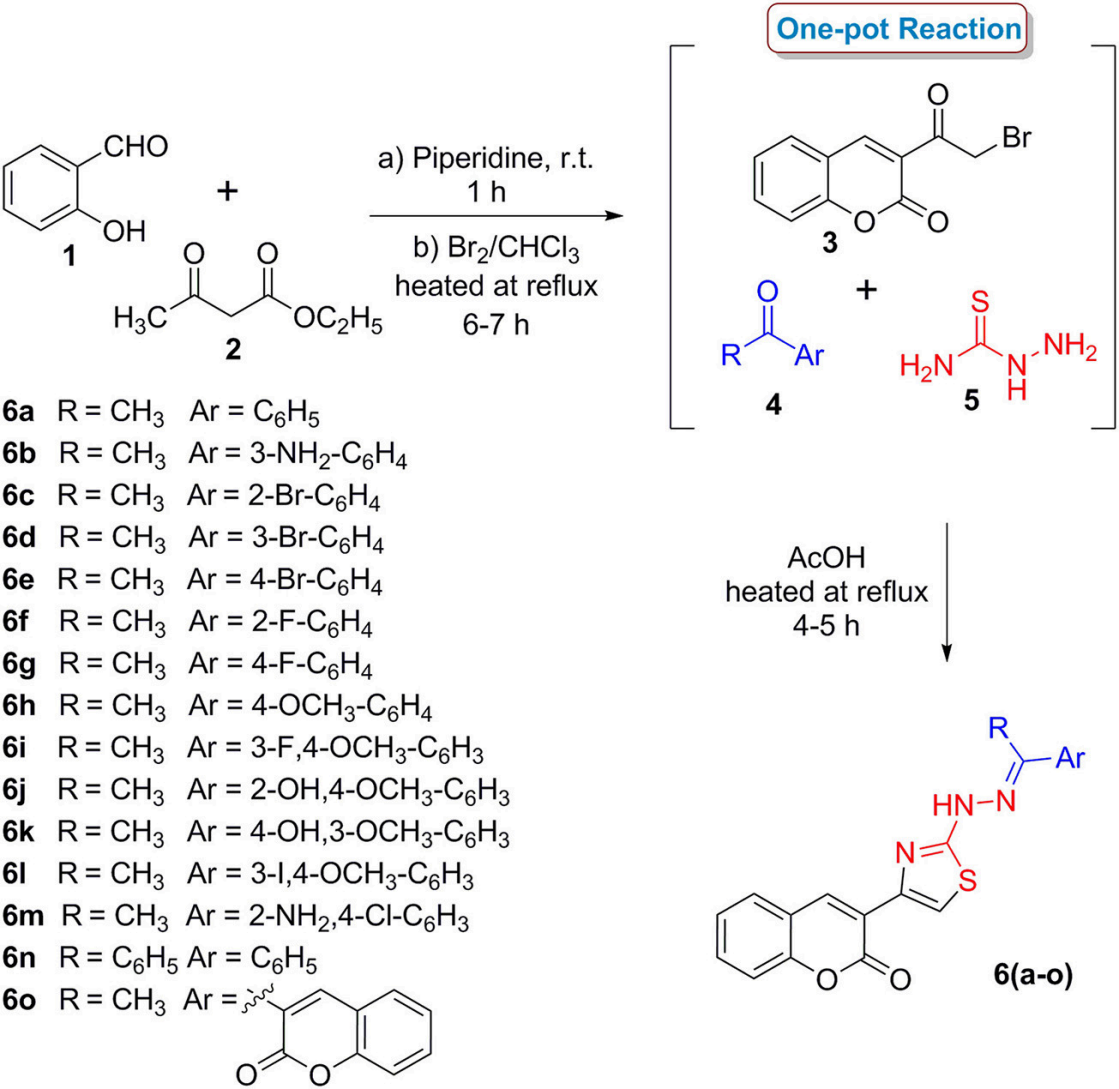
One-pot multi-component synthetic protocol for the preparation of coumarinyl thiazole derivatives **6(a–o)**.

Two series of coumarinyl thiazoles **6(a–o)** and oxadiazoles **11(a–h)** were prepared with the aim to identify new and potent inhibitors of acetylcholinesterase and butyrylcholinesterase. Coumarinyl thiazole derivatives **6(a–o)** were accessed through a multi-component reaction approach which starts with the preparation of 3-(2-bromoacetyl)-2*H*-chromen-2-one **(3)** *via* base-catalyzed condensation of readily available starting materials (salicylaldehyde and ethyl acetoacetate) followed by bromination (Scheme 1; Ibrar et al., 2016). An acid-catalyzed one-pot reaction of intermediate **3**, different substituted acetophenones **(4)** and thiosemicarbazide **(5)** provided the title compounds **6(a–o)** in good yields (Ibrar et al., 2016).

**Scheme 2:**
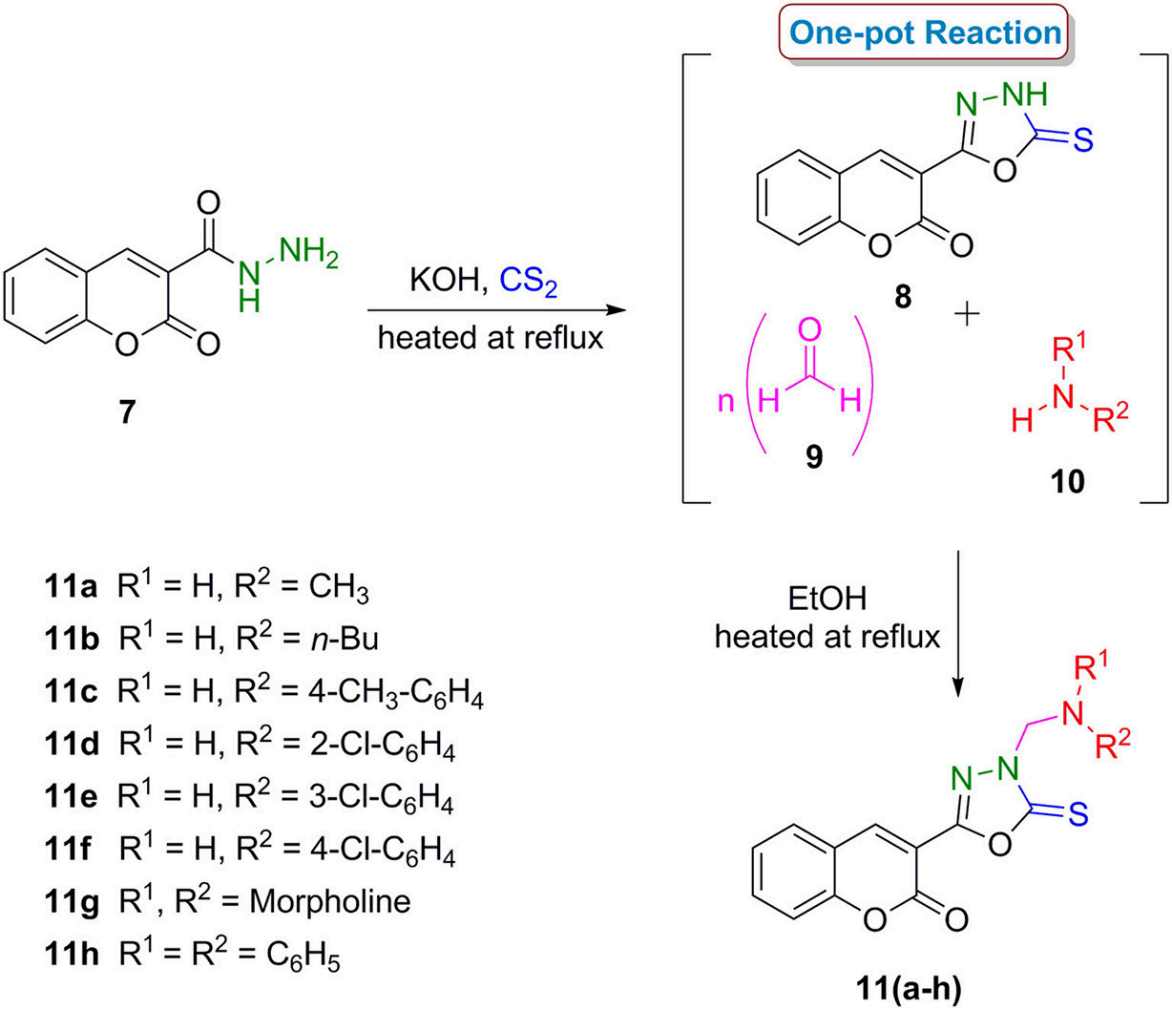
One-pot multi-component synthetic protocol for the preparation of coumarin-oxadiazole-2(3*H*)-thione conjugates **11(a–h)**.

In a second series, coumarinyl oxadiazole-2(3*H*)-thione conjugates **11(a–h)**, the central intermediate 3-(5-thioxo-4,5-dihydro-1,3,4-oxadiazol-2-yl)-2*H*-chromen-2-one **(8)** was prepared by the reaction of coumarinyl hydrazide **(7)** with carbon disulfide in ethanolic solution of KOH in good yield (Pattan et al., 2009). A one-pot reaction of compound **8**, paraformaldehyde **(9)** and different (aliphatic and aromatic) amines **(10)** gave the desired compounds **11(a–h)** in good yields (Scheme 2). The compounds were characterized by various spectroscopic techniques and full spectro-analytical data is described in our recent report (Ibrar et al., 2016).

### Pharmacology

The target compounds, coumarinyl thiazoles **6(a–o)** and coumarinyl oxadiazoles **11(a–h)**, were screened for their inhibitory activity against AChE and BuChE by Ellman's method. All the assays were carried out at micromolar level using neostigmine and donepezil as standard inhibitors having IC_50_ values of 28.2 ± 2.01 and 7.23 ± 0.13 μM for AChE, whereas 16.1 ± 1.13 and 0.03 ± 0.003 μM for BuChE, respectively. The results obtained for both series **6(a–o)** and **11(a–h)** are summarized in Tables 1, 2. The IC_50_ values revealed that most of the synthesized compounds displayed potent and selective inhibition toward cholinesterases.

**Table 1:** Inhibition potency of coumarinyl thiazoles **6(a–o)** against AChE and BuChE.

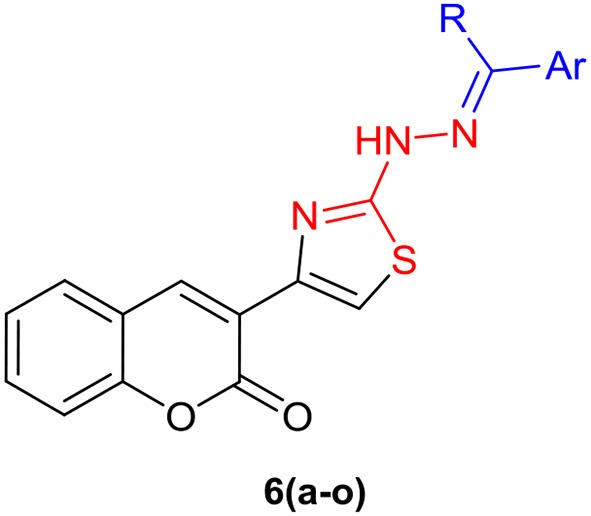						
**Compound**	**Substituent (R)**	**Substituent (Ar)**	**AChE**	**BuChE**	**Selectivity for**	
			**IC_50_ (μM) ± SEM^a^**		**AChE**	**BuChE**
**6a**	Me	Ph	16.91 ± 0.32	32.7 ± 2.08	1.93	0.51
**6b**	Me	3-NH_2_-Ph	0.87 ± 0.09	31.22 ± 0.17	35.8	0.027
**6c**	Me	2-Br-Ph	21.19 ± 0.23	19.33 ± 1.42	0.91	1.09
**6d**	Me	3-Br-Ph	30.06 ± 1.73	15.16 ± 0.53	0.50	1.98
**6e**	Me	4-Br-Ph	33.42 ± 0.18	49.12 ± 0.12	1.47	0.68
**6f**	Me	2-F-Ph	23.06 ± 0.32	32.07 ± 1.21	1.39	0.71
**6g**	Me	4-F-Ph	36.34 ± 0.18	28.01 ± 0.21	0.77	1.29
**6h**	Me	4-OMe-Ph	1.08 ± 0.84	19.32 ± 0.11	17.8	0.055
**6i**	Me	3-F-4-OMe-Ph	2.34 ± 1.43	22.1 ± 1.08	9.44	0.105
**6j**	Me	2-OH-4-OMe-Ph	9.05 ± 0.23	11.01 ± 3.37	1.22	0.81
**6k**	Me	4-OH-3-OMe-Ph	5.86 ± 0.15	19.13 ± 1.32	3.29	0.303
**6l**	Me	3-I-4-OMe-Ph	13.84 ± 1.58	32.1 ± 1.08	2.32	0.42
**6m**	Me	2-NH_2_-4-Cl-Ph	22.74 ± 0.16	37.17 ± 0.55	1.63	0.61
**6n**	Ph	Ph	11.19 ± 0.17	21.6 ± 3.09	1.94	0.51
**6o**	Me	Coumarin	10.1 ± 1.12	23.19 ± 0.29	2.28	0.43
**Neostigmine**	–	–	28.2 ± 2.01	16.1 ± 1.13	0.57	1.75
**Donepezil**	–	–	7.23 ± 0.13	0.03 ± 0.003	0.004	241

a *SEM, Standard mean error of three experiments*.

**Table 2:** Inhibition potency of coumarinyl oxadiazoles **11(a–h)** against AChE and BuChE.

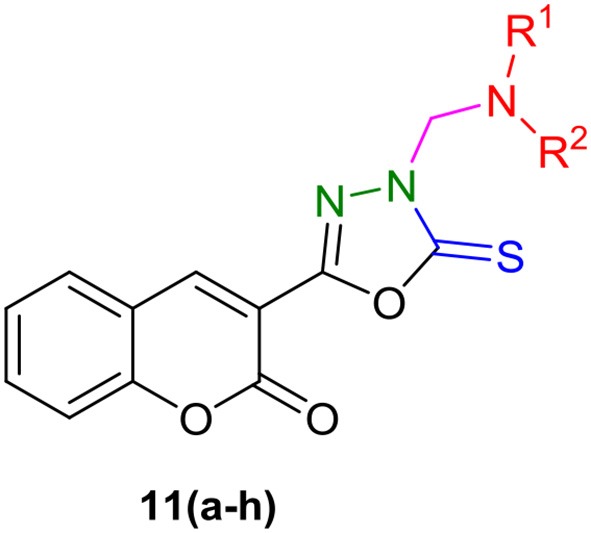						
**Compound**	**Substituent (R^1^)**	**Substituent (R^2^)**	**AChE**	**BuChE**	**Selectivity for**	
			**IC_50_ (μM) ± SEM^a^**		**AChE**	**BuChE**
**11a**	H	Me	6.07 ± 0.23	0.341 ± 0.06	0.056	17.8
**11b**	H	*n*-Bu	9.41 ± 0.55	0.77 ± 0.08	0.081	12.2
**11c**	H	4-Me-Ph	23.8 ± 0.94	8.01 ± 3.58	0.33	2.97
**11d**	H	2-Cl-Ph	16.6 ± 2.72	3.48 ± 1.58	0.20	4.77
**11e**	H	3-Cl-Ph	12.4 ± 0.08	0.15 ± 0.09	0.012	82.6
**11f**	H	4-Cl-Ph	18.6 ± 0.32	12.1 ± 1.08	0.65	1.53
**11g**	Ph	Ph	9.57 ± 1.42	4.54 ± 0.54	0.47	2.10
**11h**	Morpholine		12.2 ± 1.67	13.46 ± 0.41	1.09	0.91
**Neostigmine**	**–**	**–**	28.2 ± 2.01	16.1 ± 1.13	0.57	1.75
**Donepezil**	**–**	**–**	7.23 ± 0.13	0.03 ± 0.003	0.004	241

a *SEM, Standard mean error of three experiments*.

**Figure 2:**
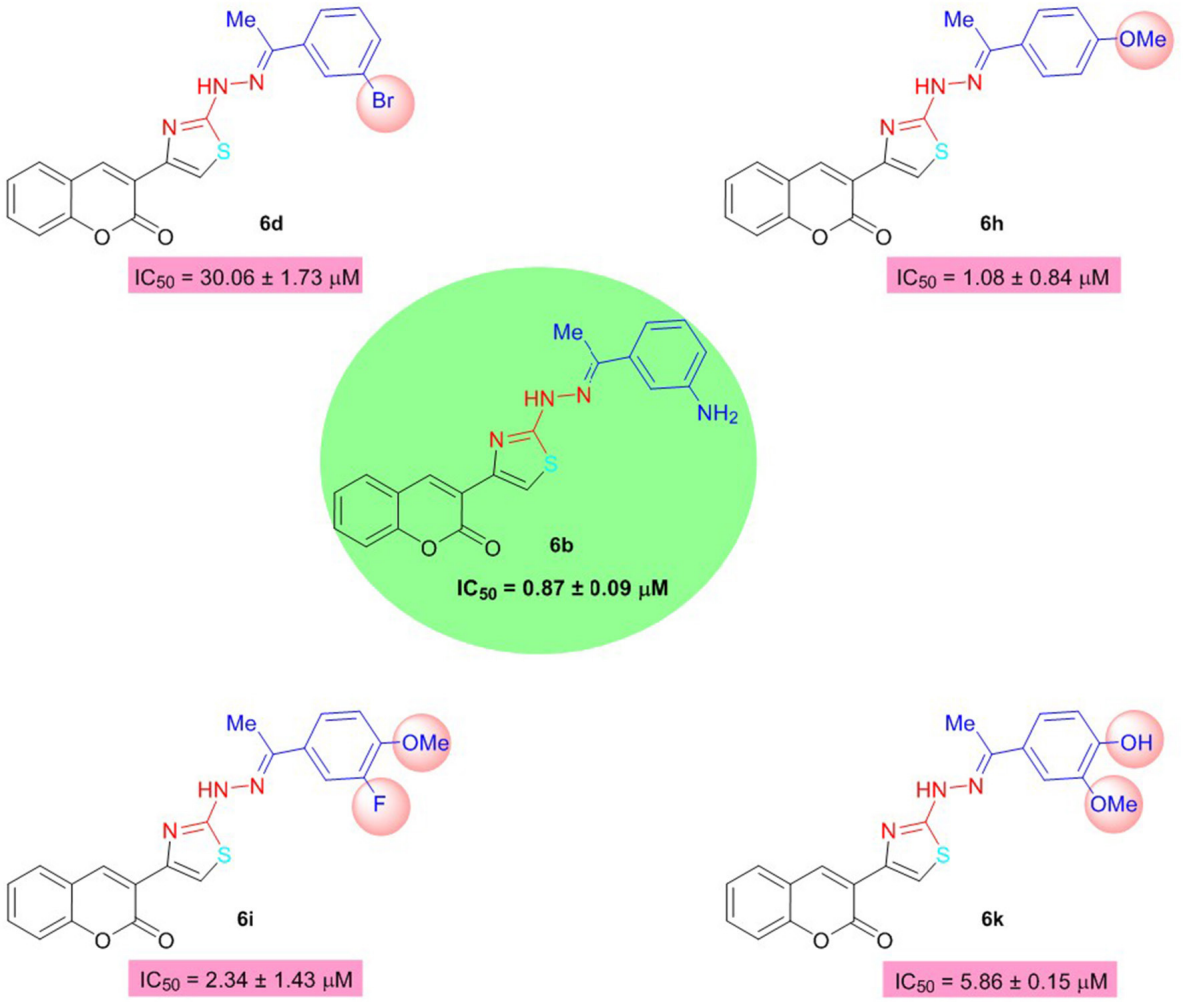
Structure-activity relationship of compound **6b** with **6d**, **6h**, **6i**, and **6k**.

Among them, **6b** of the coumarinyl thiazole series was found to be the strongest AChE inhibitor with an IC_50_ value of 0.87 ± 0.09 μM (Table 1, Figure 2). This compound inhibited AChE ~32-fold more strongly than the standard neostigmine, and nine-fold as effective against AChE as the second standard donepezil (IC_50_ = 7.23 ± 0.12 μM). The strong inhibitory potential of **6b** could be credited to the electron-donating amine group present at *meta*-position of the aryl ring. The introduction of a bromo group at the *meta*-position produced comparable results (**6d**; IC_50_ = 30.06 ± 1.73 μM) to the neostigmine. A slight decrease in the inhibition (IC_50_ = 1.08 ± 0.84 μM) was observed in case of compound **6h** having methoxy group at *para*-position but the inhibition was still 26-fold stronger than the standard neostigmine (Figure 2). However, compounds **6i** and **6k** bearing a double substitution at the aryl ring showed IC_50_ values of 2.34 ± 1.34 and 5.86 ± 0.15 μM, respectively. These compounds incorporate a combination of different electron-donating and electron-withdrawing groups which could potentially lead to increase the several folds in AChE inhibition than the neostigmine and comparable inhibition (in case of **6k**) to the donepezil (Figure 2). In the same series **(6a–o)**, a slight decrease in the inhibition was observed in compounds **6j**, **6n**, **6o**, and **6l** as compared to the potent analogs, but the inhibition was still stronger compared to neostigmine.

**Figure 3:**
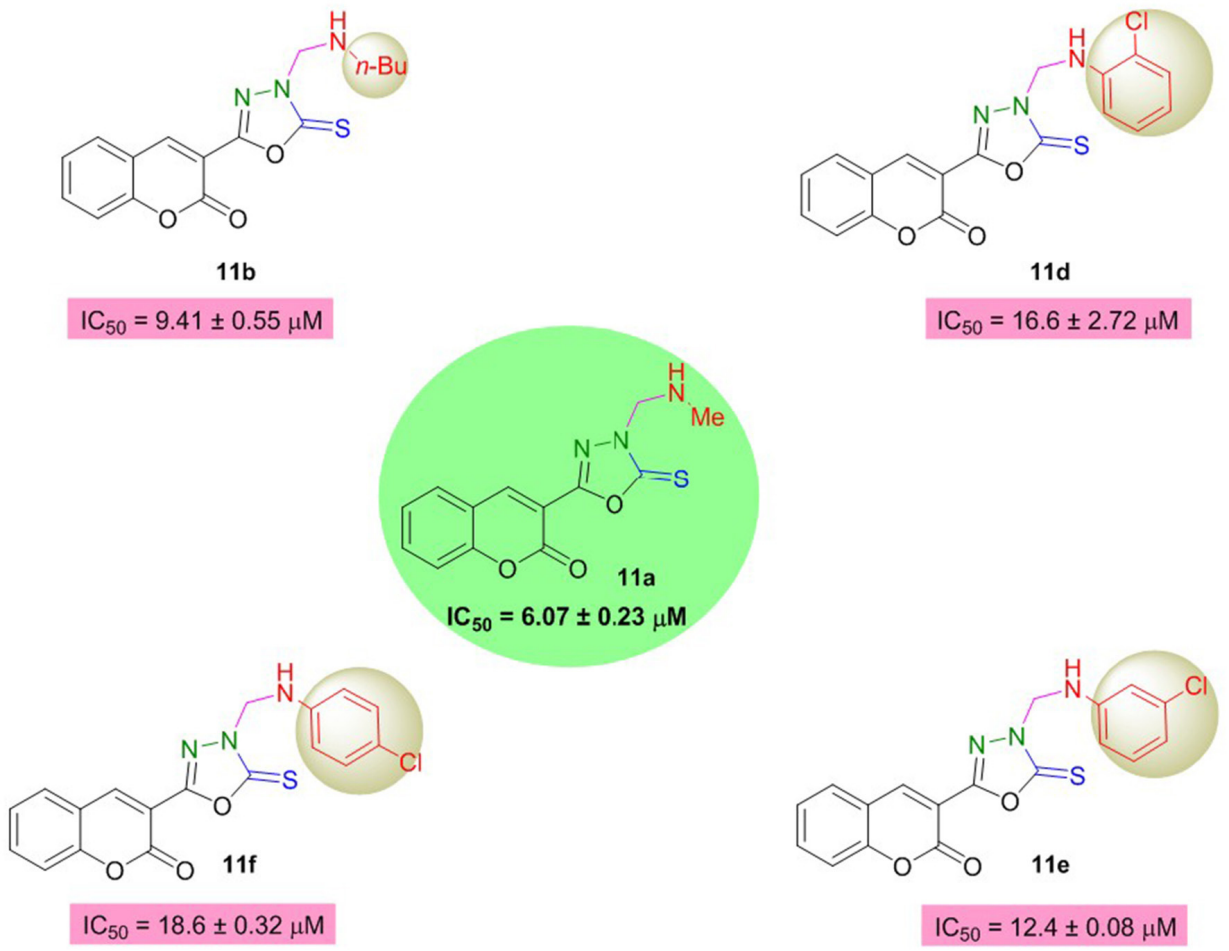
Structure-activity relationship of compound **11a** with **11b** and **11d–f**.

On the other hand, in the coumarinyl oxadiazole series **(11a–h)**, compound **11a** was found to be the most potent AChE inhibitor having IC_50_ value of 6.07 ± 0.23 μM (Table 2, Figure 3). This inhibitory potency might be attributed to an aliphatic methyl group substituted on the amine moiety. A slight decrease in the inhibition was observed when the methyl group was replaced by another aliphatic (*n*-Bu) group as revealed by compound **11b** (IC_50_ = 9.41 ± 0.55 μM). When these aliphatic groups were replaced by aromatic substitutions as in **11c–f**, reduced inhibition was observed (Figure 3).

**Figure 4:**
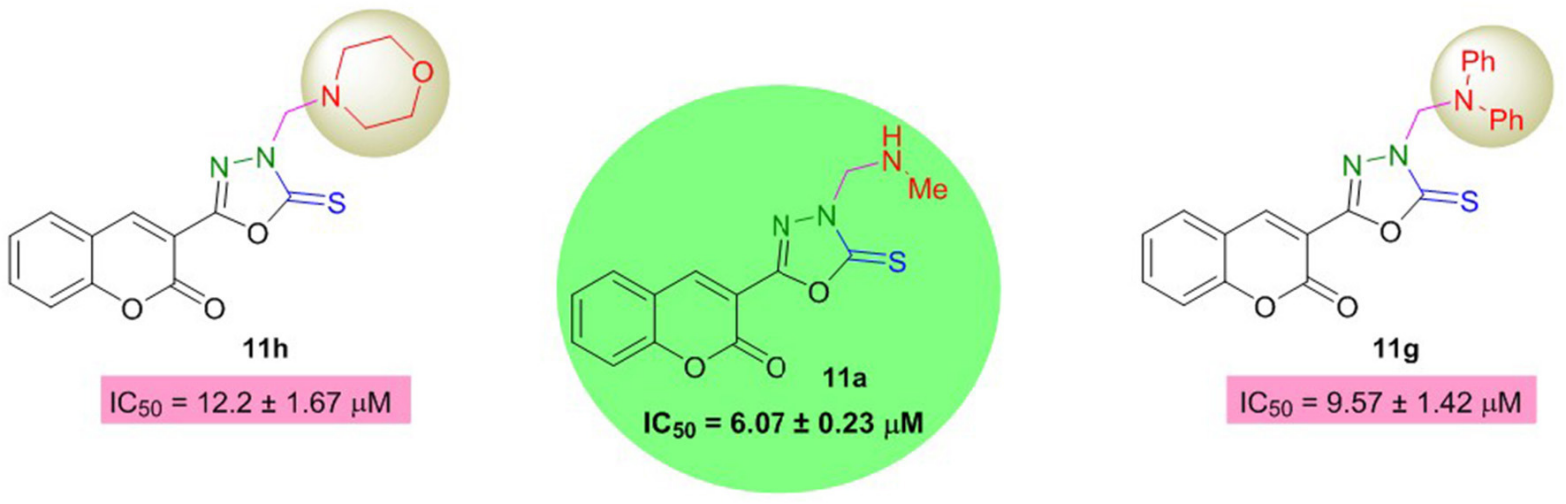
Structure-activity relationship of compound **11a** with **11g** and **11h**.

Moreover, oxadiazole compounds with morpholine substituent (**11g**) and two phenyl groups **(11h)** were also found as moderate inhibitors of AChE with two- and three-fold higher inhibition as compared to neostigmine (Figure 4). Overall, among the tested compounds, coumarinyl thiazoles appeared as potent AChE inhibitors than the coumarinyl oxadiazoles.

**Figure 5:**
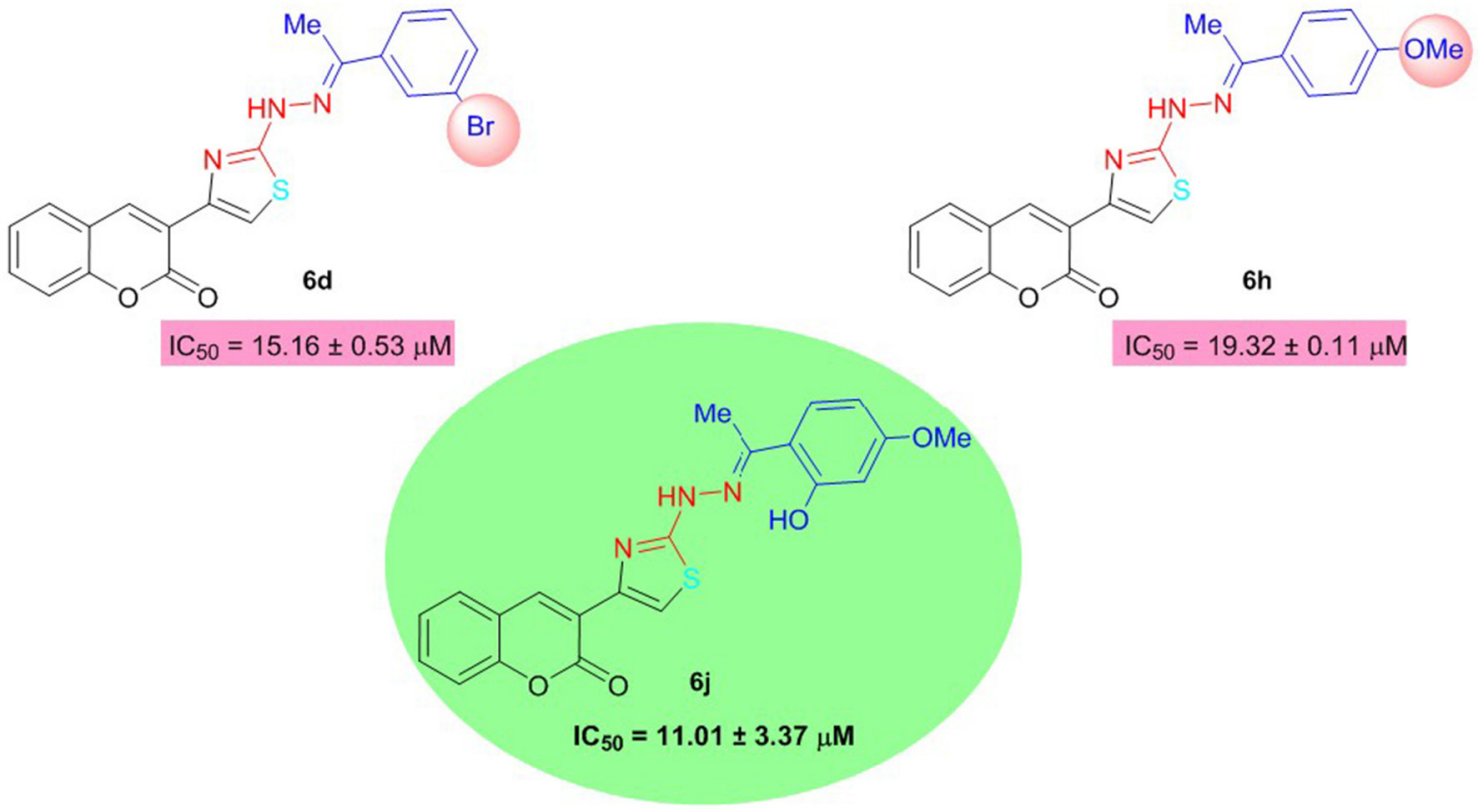
Structure-activity relationship of compound **6j** with **6d** and **6h**.

All the synthesized analogs were also evaluated for butyrylcholinesterase inhibition and several compounds were found to possess potent inhibitory activity higher than the standard neostigmine. Among the coumarinyl thiazoles, compound **6j** with dual electron-donating groups was the lead inhibitor with IC_50_ value of 11.01 ± 3.37 μM. Compounds **6d** and **6h** with *meta*-bromo and *para*-methoxy substituents were also moderate inhibitors of BuChE (Figure 5). The other compounds in the series showed weak inhibition for BuChE.

**Figure 6:**
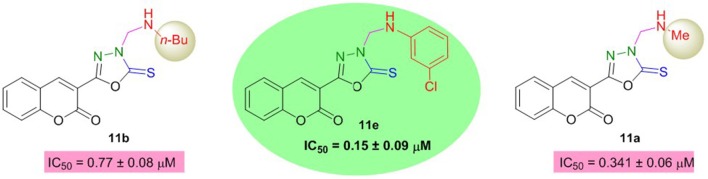
Structure-activity relationship of compound **11e** with **11a** and **11b**.

However, the coumarinyl oxadiazoles were strong inhibitors of BuChE. Compound **11e** bearing *meta*-chloro substituent inhibited the BuChE with an IC_50_ value of 0.15 ± 0.09 μM. This compound was about 107-fold more potent than neostigmine. The replacement of chloro phenyl with an aliphatic methyl and *n*-Bu group (**11a**; IC_50_ = 0.341 ± 0.06 μM, **11b**; IC_50_ = 0.77 ± 0.08 μM) directed a small decrease in the inhibition but the compounds were still several-folds more active than neostigmine (Figure 6). The other compounds in the series **11c**, **11d**, **11f**, **11h**, and **11g** revealed significant inhibition more than the reference standard.

In general, among the synthesized analogs, the compounds from coumarinyl thiazole series **(6a–o)** were excellent AChE inhibitors than the two reference drugs neostigmine and donepezil while coumarinyl oxadiazoles **(11a–h)** showed strong inhibition for BuChE than the standard neostigmine. All in all, the target analogs proved to be very potent inhibitors of cholinesterase and by considering their strong inhibitory potential, these heterocyclic hybrid compounds hold great potential for the development of new targets for AD therapy.

### Molecular docking studies

**Figure 7:**
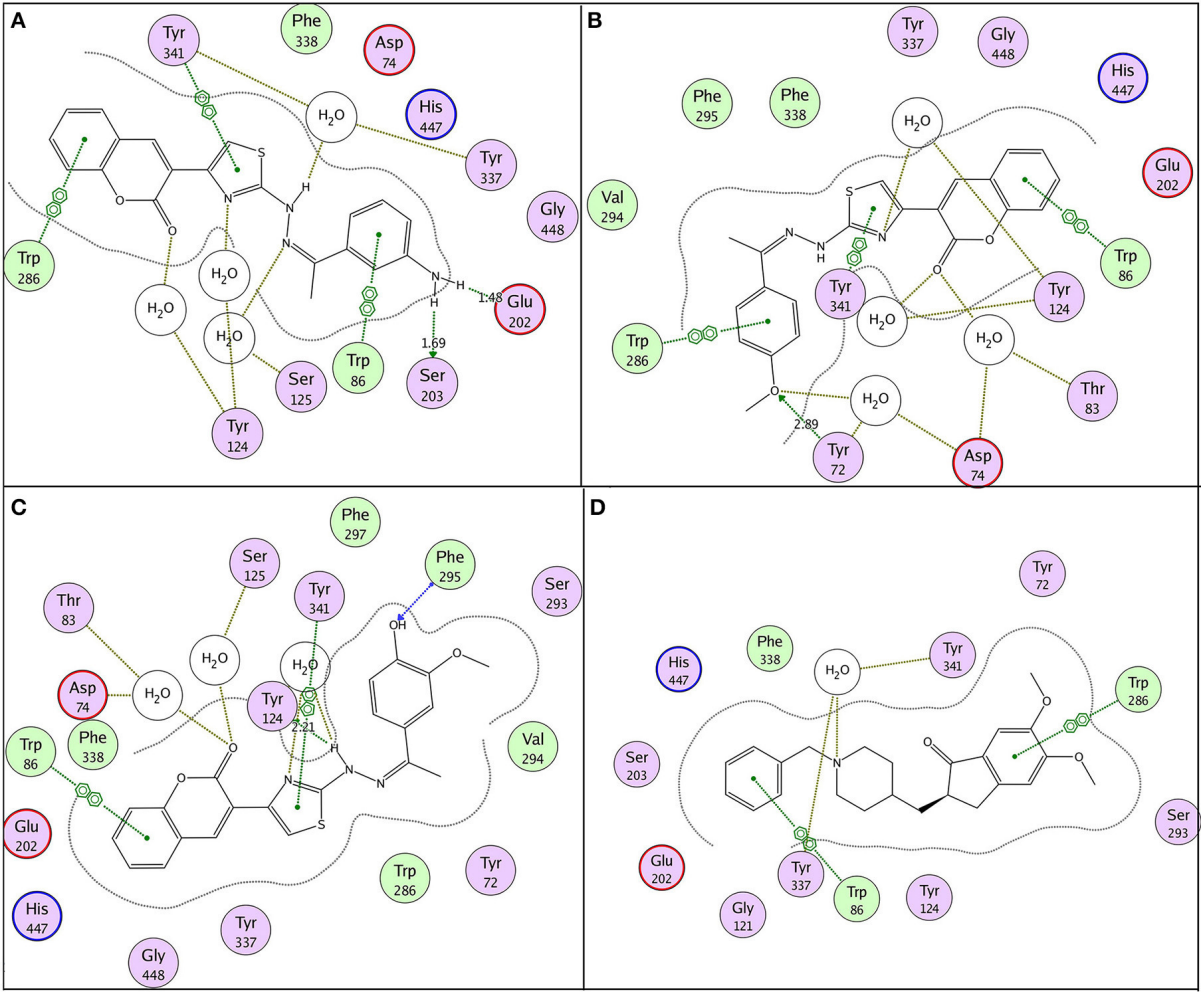
2D binding pose representation of compound **(A) 6b**, **(B) 6h**, **(C) 6k**, and **(D) donepezil** with the active site of AChE (4EY7) chain A.

**Figure 8:**
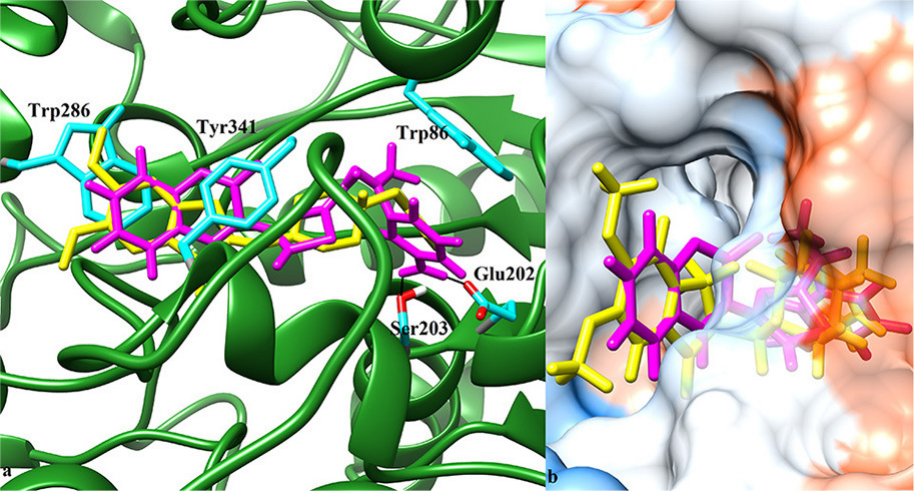
**(a)** 3D ribbon model of AChE (4EY7) chain A (green) docked with compound **6b** (magenta) and co-crystalized ligand **donepezil** (yellow). Interacting residues shown in ball and stick model (cyan). Black solid lines represent hydrogen bonding. **(b)** Hydrophobic surface model and active site cavity of the enzyme bound with donepezil (yellow) and compound **6b** (magenta).

Possible binding modes of coumarinyl thiazole and oxadiazole derivatives were explored by MOE (Molecular Operating Environment) software. Molecular docking studies revealed that the thiazoles having high anti-acetylcholinesterase activity (**6b**, **6h**, **6i**, and **6k**) represented better interaction with the active site of AChE (4EY7) with MOE score of −10.19, −9.97, −9.68, and −11.03 Kcal.mol^−1^, respectively, whereas oxadiazoles having high activity against butyrylcholinesterase (**11e**, **11a**, and **11b**) represented better interaction with BuChE (4BDS) with MOE score of −9.9, −7.4, and −8.2 Kcal.mol^−1^, respectively, as compared to reference ligands (neostigmine and donepezil) as shown in Table 3. Compound **6b** (IC_50_ = 0.87 ± 0.09 μM) demonstrated conventional hydrogen bonding with Glu202 (1.48 Å) and Ser203 (1.69 Å) due to amino group of aryl ring and π-π stacking with Trp86, Trp286, and Tyr341 along with water bridging with Tyr337, Tyr124, and Ser125 as shown in Figure 7A. The impact of amino group on the activity of compound was already mentioned earlier in the structure-activity relationship (Figure 2). Compound **6h** (IC_50_ = 1.08 ± 0.84 μM) represented hydrogen bonding with Tyr72 (2.89 Å) and π-π stacking with Trp86, Trp286, and Tyr341 and Phe338 along with water bridging with Tyr72, Tyr124, and Thr83 as shown in Figure 7B. Compound **6i** (IC_50_ = 2.34 ± 1.43 μM) also demonstrated almost similar interactions (as with compound **6h)** except the length of hydrogen bond with Tyr72 (2.93 Å). Compound **6k** represented hydrogen bond with Tyr124 (2.21 Å), π-π stacking with Trp86 and Tyr341 (not with Trp286) and water bridging with Thr83, Ser125, and Asp74 as shown in Figure 7C. Reference compound (co-crystallized ligand) donepezil was also docked to compare and confirm our docking results where it was observed that it makes only π-π stacking with Trp86 and Trp286 along with water bridging with Tyr337 and Tyr341 as shown in Figure 7D. In agreement to the *in-vitro* results, molecular docking studies suggested that compounds **6b**, **6h**, **6i**, and **6k** represented better interaction than donepezil; (i) due to hydrogen bonding, (ii) due to extra π-π stacking with aromatic residues, and (iii) due to more interaction through water bridging as shown in Figure 7. Active site of the enzyme ribbon model (4EY7) and molecular docking comparison of the most active compound **6b** (magenta) with reference ligand donepezil (yellow) was depicted in Figure 8a, whereas hydrophobic surface and active site cavity of the enzyme docked with compound **6b** (magenta) was represented in Figure 8b.

**Table 3:** MOE score of highly ranked coumarinyl thiazole and oxadiazole derivatives with the active site of AChE and BuChE.

**Compound**	**MOE score (Kcal.mol^−1^) with AChE (4EY7)**	**Compound**	**MOE score (Kcal.mol^−1^) with BuChE (4BDS)**
**6b**	−10.19	**11e**	−9.9
**6h**	−9.97	**11a**	−7.4
**6i**	−9.68	**11b**	−8.2
**6k**	−11.03	**11d**	−7.0
**Neostigmine**	−7.03	**Neostigmine**	−5.05
**Donepezil**	−9.14	**Donepezil**	−7.75

**Figure 9:**
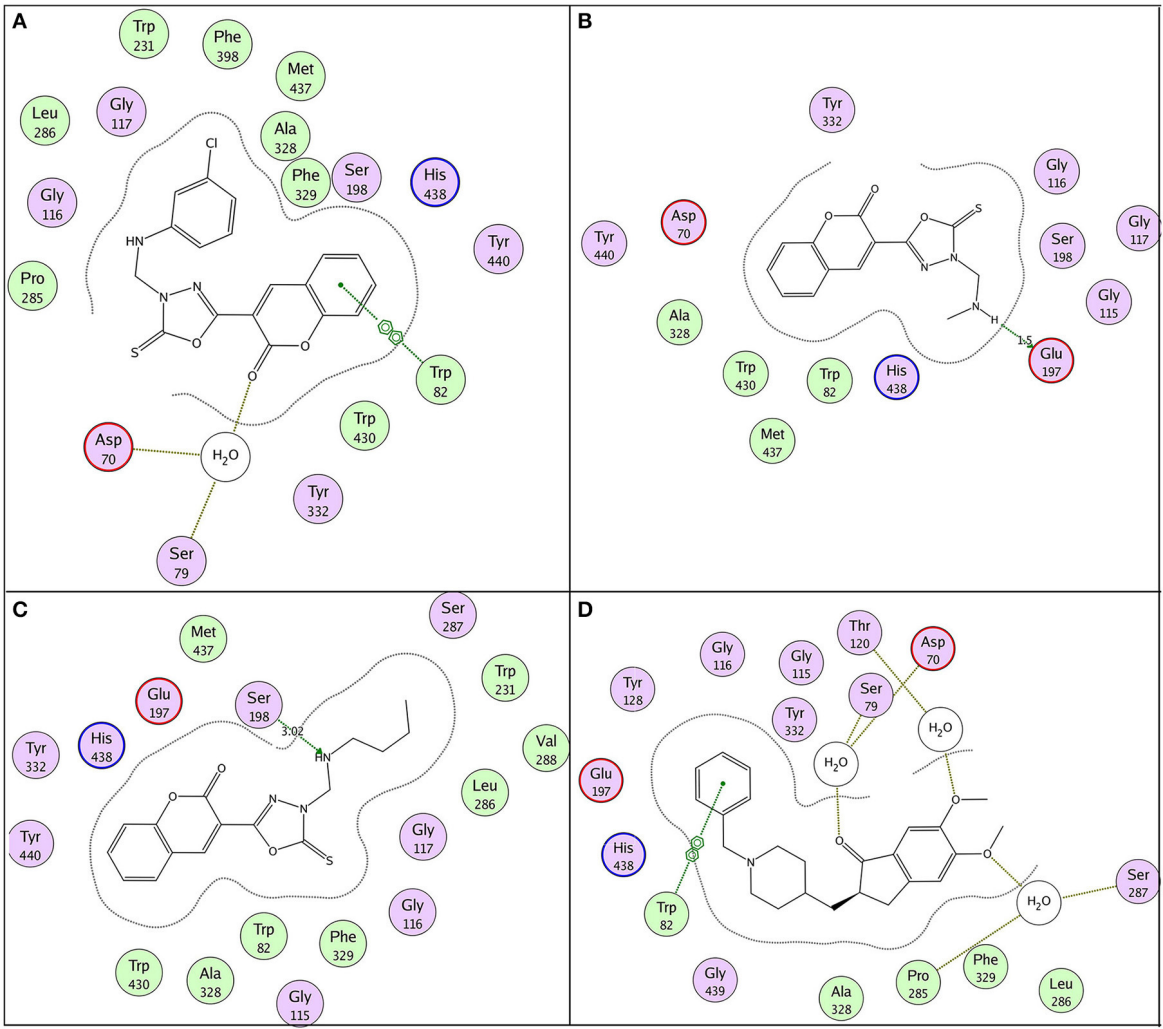
2D binding pose representation of compound **(A) 11e**, **(B) 11a**, **(C) 11b**, and **(D) donepezil** with the active site of BuChE (4BDS).

**Figure 10:**
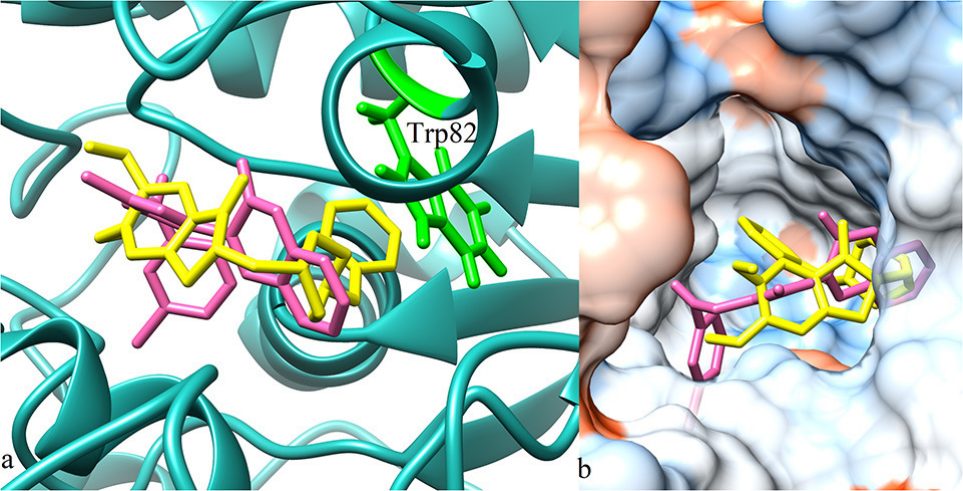
**(a)** 3D ribbon model (blue) of BuChE (4BDS) docked with compound **11e** (pink) and reference ligand **donepezil** (yellow). **(b)** Hydrophobic surface and active site cavity of the enzyme bound with donepezil (yellow) and compound **11e** (pink).

According to molecular docking results, the highest ranked anti-butyrylcholinesterase compound **11e** (IC_50_ = 0.15 ± 0.09 μM) illustrated π-π stacking with Trp82 and interaction through water bridging with Asp70 and Ser79 as shown in Figure 9A. Compound **11a** (IC_50_ = 0.34 ± 0.06 μM) and **11b** (IC_50_ = 0.77 ± 0.08 μM) represented conventional hydrogen bonding with Glu197 (1.5 Å) and Ser198 (3.0 Å), respectively as shown in Figures 9B,C. However their low affinity binding poses also demonstrated π-π stacking with Trp82 and water bridging. Reference ligand donepezil represented π-π stacking with Trp82 and interaction through water bridging with Asp70, Ser79, Thr120, Ser287, and Pro285 as shown in Figure 9D. Docking comparison of compound **11e** (pink) with reference ligand donepezil (yellow) in the active site of BuChE (4BDS) ribbon model and surface model was depicted in Figures 10a,b, respectively.

### Pharmacokinetics prediction

Early prediction of *in-silico* ADMET properties of lead molecules has now realized as an effective tool in the drug discovery and development process. Therefore, Lipinski's criteria and oral rat LD_50_ value were estimated for top ranked active compounds by using TEST (Toxicity Estimation Software Tool) and Molinspiration online software. Top **five** compounds selected from coumarinyl thiazoles and oxadiazole (6b, 6h, 6i, 11a, and 11e) for the analysis and results were summarized in Table 4. Polar surface area (tPSA) values are important for the determination of blood brain barrier (BBB) penetration. According to Waterbeemd the cutoff value is 90 Å^2^ or less. Almost all the compounds fulfilled the criteria except compound 6b with cutoff value slightly higher i.e., 93. Number of rotatable bonds (nROT) is an additional property that measures the flexibility of the molecule. The drugs that are BBB +ve, usually reported to have fewer nROT bonds. Another extension in RO5 to improve the prediction of drug-likeness is molar refractivity (MR) which should be 40–130. Oral rat LD_50_ was also predicted for these compounds and the compounds were found slightly toxic according to Hodge and Sterner scale. All the criteria were fulfilled by the compounds and no Lipinski's violation was found as shown in Table 4.

**Table 4:** Pharmacokinetics prediction of top ranked compounds.

**Compound**	**MW**	**LogP**	**MR**	**HBD**	**HBA**	**nROT**	**tPSA**	**BBB**	**LD_50_**
**6b**	376.5	3.5	109	3	6	4	93	−ve	937.3
**6h**	391.4	4.5	110	1	6	5	77	+ve	523.0
**6i**	409.5	5.1	111	1	6	5	77	+ve	1,188
**11a**	289.3	1.4	77	1	6	3	73	+ve	N/A
**11e**	385.8	3.79	103	1	6	4	73	+ve	N/A

## Conclusions

In summary, the present report clearly revealed that the new hybrid molecules show remarkable inhibition of AChE and BuChE enzymes. Compound **6b** from coumarinyl thiazole series was emerged as the most potent inhibitor of AChE, whereas **11e** from coumarinyl oxadiazole derivatives inhibited the BuChE with highest potency. Both the identified inhibitors follow Lipinski's RO5, slightly toxic and near the range of blood brain barrier crossing. In future, these compounds and their functionalized derivatives may be helpful in the development of potent drugs for Alzheimer's disease.

## Experimental

### Synthesis of coumarinyl thiazole 6(a–o) and oxadiazole 11(a–h) derivatives

The coumarinyl thiazole **6(a–o)** and oxadiazole **11(a–h)** analogs were prepared according to our recently published report (Ibrar et al., 2016).

### Pharmacological protocols

#### Methodology for determining AChE and BuChE inhibitory activity

For the determination of cholinesterase inhibition, electric eel, and horse serum were used as source of AChE and BuChE, respectively. The Ellman's spectrophotometric method was used to determine the AChE and BuChE inhibitory activity with a slight modification (Ellman et al., 1961). The compounds with 1 Mm concentration were prepared in DMSO. Assay was carried out in 96 well-plate in triplicates. The reaction mixture comprised of 20 μL of buffer (tris HCl 50 mM, 0.02 M MgCl_2_.6H_2_O, 0.1 mM NaCl) at pH 8, 10 μL of the test compound, 10 μL enzyme acetylcholine or butyrylcholinesterase of 0.03 U/mL (500 U of AChE and 700 U/mg of BuChE). The contents were incubated for 10 min at 25°C followed by the addition of 1 mM of 10 μL of substrate acetylcholine iodide for AChE and butyrylthiocholine iodide for BuChE and incubated again at 25°C for 15 min. A 50 μL of 3 mM DTNB as a coloring agent was added and incubated at 25°C for further 10 min. The amount of product formed was measured by using micro plate reader (Bio-Tek ELx 800, Instruments Inc., Winooski, VT, USA) at 405 nm. The enzyme dilutions were made by using buffer of pH 8 (tris base 50 mM and having 0.1% BSA). The compounds which depict inhibitory activity more than 50% were further tested by making 9–12 serial dilutions in assay buffer and IC_50_ values were calculated by graph pad prism.

### Molecular docking

The molecular construction of the compounds was performed using ChemBioDraw Ultra 14 suite (PerkinElmer Inc.) and converted into 3D conformations by ChemBio3D (Mills, 2006). Molecular docking studies of the compounds were carried out using MOE (Molecular Operating Environment) software (ChemicalComputingGroup, 2008). The structures of the compounds were energy minimized using MMFF94x forcefield and gradient: 0.05. Crystal structures of the enzymes, acetylcholinesterase (PDB: 4EY7) and butyrylcholinesterase (PDB: 4BDS) were retrieved from Protein Data Bank (Berman et al., 2006). The co-crystallized ligands in the active site of AChE and BuChE, donepezil (PDB: E20), and Tacrine (PDB: THA) were taken as possible binding site. Ligand neostigmine was taken from PubChem (CID:4456) (Kim et al., 2015). The target proteins were prepared by the addition of hydrogen. All other parameters were used with the default settings. Donepezil and neostigmine were taken as reference ligands for comparison purposes. For each ligand 10 conformations were generated. The images in 2D were captured through MOE ligand binding interaction. 3D images were taken using UCSF Chimera 1.11 software (Pettersen et al., 2004).

## Author contributions

All authors listed have made a substantial, direct and intellectual contribution to the work, and approved it for publication.

### Conflict of interest statement

The authors declare that the research was conducted in the absence of any commercial or financial relationships that could be construed as a potential conflict of interest.
